# Blind-Sight vs. Degraded-Sight: Different Measures Tell a Different Story

**DOI:** 10.3389/fpsyg.2016.00901

**Published:** 2016-06-16

**Authors:** Chiara Mazzi, Chiara Bagattini, Silvia Savazzi

**Affiliations:** ^1^University of Verona and National Institute of NeuroscienceVerona, Italy; ^2^Perception and Awareness (PandA) Laboratory, Department of Neuroscience, Biomedicine and Movement Sciences, University of VeronaVerona, Italy; ^3^Cognitive Neuroscience Section, IRCCS Centro San Giovanni di Dio FatebenefratelliBrescia, Italy

**Keywords:** visual awareness, blindsight, primary visual cortex, perceptual awareness scale, degraded vision

## Abstract

Blindsight patients can detect, localize, and discriminate visual stimuli in their blind field, despite denying being able to see the stimuli. However, the literature documents the cases of blindsight patients who demonstrated a preserved degree of awareness in their impaired visual field. The aim of this study is to investigate the nature of visual processing within the impaired visual field and to ask whether it reflects pure unconscious behavior or conscious, yet degraded, vision. A hemianopic patient (SL) with a complete lesion to the left primary visual cortex was tested. SL was asked to discriminate several stimulus features (orientation, color, contrast, and motion) presented in her impaired visual field in a two-alternative forced-choice task. SL had to report her subjective experience: in the first experiment as “seen” or “guessed,” whereas in the second experiment as the degree of clarity of her experience according to the perceptual awareness scale. In the first experiment, SL demonstrated a performance above-chance in the discrimination task for “guessed” trials, thus showing type 1 blindsight. In the second experiment, however, SL showed above-chance performance only when she reported a certain degree of awareness, thus showing that SL’s preserved discrimination ability relies on conscious vision. These data show that graded measures to assess awareness, which can better tap on the complexity of conscious experience, need to be used in order to differentiate genuine forms of blindsight from degraded conscious vision.

## Introduction

Patients with a destruction or disconnection of all or some parts of the striate cortex (area V1) experience a region of blindness (a scotoma) in the corresponding portion of the visual field (Holmes, 1945). However, some patients, when forced to do so, can detect, localize, and discriminate stimuli briefly presented in their impaired visual field despite denying being able to see the stimuli (Pöppel et al., 1973; Weiskrantz et al., 1974). For this phenomenon, the oxymoron “blindsight” was coined (Weiskrantz et al., 1974) to highlight the dissociation between conscious perception (absent) and performance (good). Indeed, the term “blind” refers to the patient’s self-reports of not being able to see the stimulus in her/his blind field, while “sight” is demonstrated by her/his above-chance performance in binary forced-choice tasks where the patient is requested to “guess” a specific characteristic of the stimulus she/he denies seeing (Cowey, 2010).

Despite being considered by some researchers as an artifact (e.g., Campion et al., 1983), blindsight remains, after 40 years of investigation, one of the most striking example of visual processing in the absence of visual awareness. This extensive investigation, indeed, has revealed the large variety of features (e.g., motion, orientation, and color), that can be successfully processed by the brain and affect behavior in the absence of visual awareness (for review, see Stoerig and Cowey, 1997; Stoerig, 2006). Recently, the properties of stimuli that can be unconsciously processed by blindsight patients have been found to extend beyond simple features (Tamietto and Morrone, 2016), such as including categorical perception (Trevethan et al., 2007; Van den Stock et al., 2014), face identity or familiarity (Solcà et al., 2015), facial or bodily expression (for a recent review, see Celeghin et al., 2015a). Over the years, however, the definition of blindsight has changed and two forms of it have been described (Weiskrantz, 1998): type 1 blindsight is the classical type in which the patient reports no awareness of any kind, and type 2 blindsight where the patient reports the feeling that something has occurred in the blind field but denying any perceptual awareness of it. That is, with type 2 blindsight, the patient is aware that something has happened in her/his blind field but lacks visual qualia, thus remaining unconscious to the phenomenical contents of the visual stimulus.

Type 2 blindsight is clearly different from other forms of awareness of stimuli in the blind field, such as the Riddoch syndrome (Riddoch, 1917) where patients are conscious of having seen movement in their blind field without being able to report any other feature of the stimuli. In the Riddoch syndrome, patients report having visual qualia whereas, by definition, in type 2 blindsight visual qualia are absent. A few authors (Zeki and Ffytche, 1998; Stoerig and Barth, 2001), however, interpreted the feelings reported by these patients not as non-visual in origin, such as pertaining to the abstract knowledge of the occurrence of the stimulus, but as weak, low-level visual experiences. If this interpretation is correct, type 2 blindsight would be better described as conscious but degraded vision instead of blindsight. Indeed, the term blindsight would not be applicable as the patient is not blind. A clear example of this debate can be found in studies investigating the visual abilities of patient GY, one of the most extensively studied blindsight patients. GY reports awareness of fast motion and high-contrast visual targets in his blind visual field. This self-report has been interpreted by some (Weiskrantz et al., 1999) as evidence of non-visual knowledge of motion whereas by others (Zeki and Ffytche, 1998; Stoerig and Barth, 2001) as evidence of weak visual experiences. It must, however, be noted that GY’s descriptions of that “feeling” changed over time (Macpherson, 2015), thus rendering it difficult to reach a consensus, at least with respect to the nature of his feeling.

The controversy over the visual vs. non-visual nature of awareness in the blind field of type 2 blindsight patients is, in fact, not an easy one to solve, as only the patients’ verbal reports can be taken into account. In this respect, it has been suggested (Ramsøy and Overgaard, 2004) that the task used to assess awareness in these patients might be too crude (“yes/no” binary responses) to grasp subtle differences in phenomenal contents. To obtain a finer measure of perceptual awareness, the authors developed the perceptual awareness scale (PAS) by asking healthy participants to spontaneously scale the clarity of their perceptual experiences. They all consistently used a four-point scale to make their perceptual judgments: (1) no experience of the stimulus, (2) brief glimpse, (3) almost clear experience, and (4) clear experience. Recent magnetoencephalography (Andersen et al., 2015) and electroencephalography (Tagliabue et al., 2016) studies have found that the PAS showed a strong correlation between performance and awareness both on behavioral and neural levels and can thus better reveal the complexity and continuous nature of perceptual awareness than dichotomous measures. Overgaard et al. (2008) tested a “blindsight” patient with both a dichotomous measure (yes–no responses) and the PAS. The authors asked patient GR to identify visual stimuli (letters or geometrical figures) briefly presented in her blind field (the upper-right quadrant) and they found divergent results depending on the measure used. With the dichotomous measure (“yes/no” response), the patient appeared to display type 1 blindsight, i.e., accurate behavior in the absence of acknowledged perceptual awareness. However, when GR was asked to classify the clarity of her perception with the PAS, she reported to have seen more stimuli than in the previous task. Her criterion to acknowledge awareness changed and her performance was accurate only for stimuli she reported to have seen.

This evidence, which, to the best of our knowledge, is the only one of its kind, is very important as it demonstrates that a finer measure to assess perceptual awareness, i.e., the PAS, can better substantiate the continuous nature of awareness as far as visual qualia are concerned. Unfortunately, these results cannot, in our opinion, be considered conclusive for various reasons. First, the task at hand (discrimination of letters or geometrical figures) is not the typically used with blindsight patients. Second, and more importantly, the number of trials administered to the patient was very low (33), the stimuli were presented in different locations (11) and with different durations (3), thus possibly rendering the probability to fall within a specific level of perceptual scales (between the “seen” and “unseen” categories or among the four levels of the PAS) unequal. It is possible that “unseen” responses refer only to stimuli presented for shorter durations at a less preserved visual field locations, whereas the “seen” responses (whether in terms of the single category in the dichotomous response or the three different positive awareness categories in the PAS) only refer to stimuli presented longer at a more preserved location. This paper, thus, aims to further investigate perceptual awareness in the blind visual field by extending the investigation by Overgaard et al. (2008) while taking into account possible confounds induced by the task and stimuli used. In this study, we used both dichotomous and graded measures of awareness and asked the patient to discriminate several stimulus features such as orientation, color, contrast, and apparent and real motion, i.e., the classical features used with blindsight patients. Moreover, an appropriate number of trials was administered to the patient, and the stimuli were always presented at the same location and with the same duration. Finally, to account for possible spurious effects on the patient’s performance, we used a control condition, such as stimuli that cannot be discriminated even by sighted participants.

## Experiment 1

### Materials and Methods

#### Participants

**FIGURE 1:**
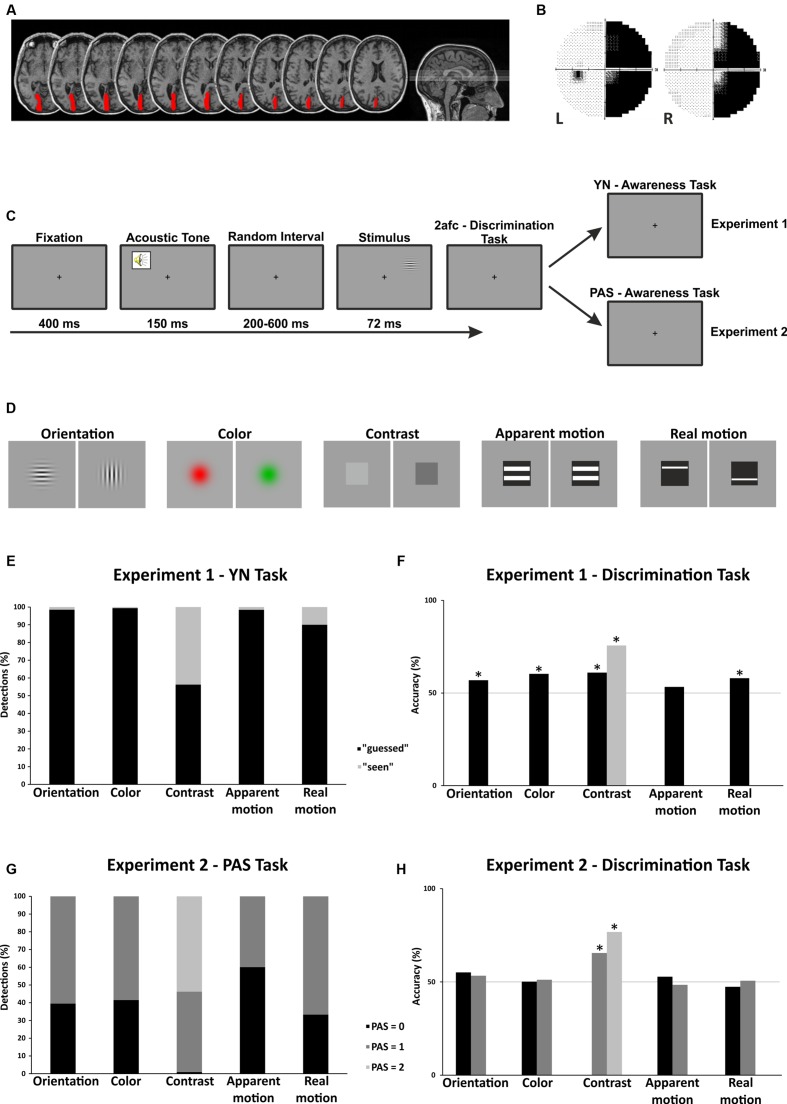
**(A)** Brain lesion reconstruction. **(B)** Visual field defect. **(C)** Experimental procedure. **(D)** Example of the stimuli used. **(E)** Experiment 1. Results of the YN awareness task as a function of the stimulus features. **(F)** Experiment 1. Results of the 2AFC discrimination task as a function of the stimulus features. **(G)** Experiment 2. Results of the PAS awareness task as a function of the stimulus features. The percentage of trials responded as PAS = 0 (black bars), PAS = 1 (dark gray bars) and PAS = 2 (light gray bars) is reported. **(H)** Experiment 2. Results of the 2AFC discrimination task as a function of the stimulus features. Accuracy for trials responded as PAS = 0 (black bars), PAS = 1 (dark gray bars) and PAS = 2 (light gray bars) is reported.

Patient SL is a 45-years old right-handed woman who suffered from a right homonymous hemianopia resulting from an ischemic stroke with hemorrhagic evolution. MRI (**Figure 1A**) and fMRI (see patient P5 in Celeghin et al., 2015b) documented a complete destruction of the left primary visual cortex (V1). Visual field defect (**Figure 1B**) was assessed by means of computerized perimetry (Humphrey system). The patient was tested in 2014, about 65 months after her neurological event. SL signed the informed consent prior to participating in the study and was free to withdraw at any time. The study was approved by the local Ethics Committee and conducted in accordance with the 2013 Declaration of Helsinki.

#### Apparatus and Stimuli

Patient SL was tested in a dimly lit room while sitting in a comfortable chair. An adjustable chin and forehead rest minimized her head movements and ensured that distance from the monitor remained constant at 57 cm. Visual stimuli were presented using E-prime 1.1 (Psychology Software Tools) via a 17-inch IBM G96 CRT monitor refreshing at 85 Hz (resolution 1280 × 1024 pixels). On-line monitoring of SL’s eye movements was performed by an infrared camera in order to verify that she maintained fixation during the stimulus presentation.

Different stimulus features (**Figure 1D**) were tested in five different experimental sessions, administered over five different days: *orientation* (vertical | horizontal Gabor patches. Spatial frequency = 1.4 c/°. Mean luminance = 4.89 cd/m^2^), *color* (red | green Gaussian circles. Color coordinates: red: *x* = 0.6150, *y* = 0.3526; green: *x* = 0.3135, *y* = 0.6052. Luminance = 15.07 cd/m^2^), *contrast* (light | dark gray circles. Luminance: light gray = 9.64 cd/m^2^; dark gray = 0.14 cd/m^2^. Weber contrast = 0.97) and *apparent* and *real motion direction* (upward | downward gratings and single bars. Speed of motion: grating = 33.33°/s; bar = 16.67 °/s. Mean luminance: 4.89 cd/m^2^). Each experimental session, in which only one stimulus feature was tested, was composed of eight blocks of 40 trials each (20 trials per stimulus type; e.g., for *orientation*, 10 vertical and 10 horizontal Gabor patches), for a total of 320 trials (e.g., for *orientation*, 160 vertical and 160 horizontal Gabor patches). With the exception of moving gratings and bars which were directly generated using custom-made e-Prime scripts, the other stimuli were generated using Matlab R2009a (The MathWorks Inc., Natick, MA, USA). Each stimulus subtended 4° × 4° of the visual angle and was presented for 72 ms. Stimuli were presented unilaterally in the blind (right) visual field of patient SL, and were placed 7° above and 12° lateral to the central fixation point. The location of the stimuli was tailored to SL’s visual field defect, according to her latest Humphrey’s computerized perimetry (**Figure 1B**). The background had a luminance of 4.89 cd/m^2^, except for the color session in which the luminance was 15.07 cd/m^2^.

#### Experimental Procedure

**Figure 1C** illustrates the experimental procedure. Each trial started with the appearance of a central fixation point (400 ms) which lasted throughout the entire trial. Stimulus presentation was preceded by a 1000 Hz warning acoustic tone lasting 150 ms. The interval between the warning tone and stimulus onset was randomized between 200 and 600 ms to avoid expectation. After the stimulus disappeared, SL was asked to report the stimulus feature in a two alternative forced-choice task (2AFC). When discrimination was not possible, she was requested to guess. SL was then asked to report with a binary response whether she perceived the stimulus’ feature or had guessed. In order not to bias her response criterion, patient SL was informed that a stimulus was presented in each trial (i.e., no catch trials were presented).

#### Statistical Analysis

For each of the stimulus features, trials were classified off-line on the basis of “seen” and “guessed” responses, and the percent accuracy for the feature discrimination was calculated. Accuracy, in the “seen” class of responses, indicates SL’s ability to discriminate the feature under the aware condition, while accuracy in the “guessed” class of responses, indicates her ability to discriminate the feature under the unaware condition. To assess whether SL’s performance was significantly higher than chance level (50%), we adopted the one-tailed binomial test. Two binomial tests were performed, one for the “seen” responses and one for the “unseen” responses.

### Results

#### Accuracy: Seen–Guessed Task

**Figures 1E,F** show SL’s detection rate and accuracy in discriminating stimulus features under the conscious (i.e., “seen” reports) and unconscious (i.e., “guessed” reports) conditions. Statistical analyses are reported separately for each stimulus feature.

##### Orientation discrimination

Patient SL reported to have guessed the orientation (vertical | horizontal) of the stimuli in almost all of the trials (98.75%) and to have seen the stimulus orientation only in 1.25% of the trials. Given the very low number of trials responded under the aware condition (i.e., “seen” reports), these data were not further analyzed. Under the unaware condition (i.e., “guessed” reports), SL’s accuracy in orientation discrimination (56.91%) was significantly higher than chance level (*p* < 0.01) demonstrating implicit processing of stimulus orientation.

##### Color discrimination

Patient SL reported to have guessed the color (red | green) of the stimuli in almost all of the trials (99.38%) and to have seen the stimulus color only in 0.62% of the trials. Given the very low number of trials responded under the aware condition (i.e., “seen” reports), these data were not further analyzed. Under the unaware condition (i.e., “guessed” reports), SL’s accuracy in color discrimination (60.36%) was significantly higher than chance level (*p* < 0.001), demonstrating implicit processing of stimulus color.

##### Contrast discrimination

Patient SL reported to have guessed the contrast (light | dark gray) of the stimuli in 56.25% of the trials and to have seen the stimulus contrast in the remaining 43.75% of the trials. Under the unaware condition (i.e., “guessed” reports), SL’s accuracy in contrast discrimination (61.04%) was significantly higher than chance level (*p* < 0.005) demonstrating implicit processing of stimulus contrast. Similarly, also under the aware condition (i.e., “seen” reports), SL’s accuracy (75.67%) was significantly higher than chance level (*p* < 0.001), demonstrating explicit processing of stimulus contrast.

##### Grating – (apparent) motion direction discrimination

Patient SL reported to have guessed the motion direction (upward | downward) of gratings in almost all of the trials (98.44%) and to have seen the motion direction only in 1.56% of the trials. Given the very low number of trials responded under the aware condition (i.e., “seen” reports), these data were not further analyzed. Under the unaware condition (i.e., “guessed” reports), SL’s accuracy in motion direction discrimination (53.31%) was not significantly higher than chance level (*p* = 0.130), demonstrating no implicit processing of motion direction of the gratings.

##### Bar – (real) motion direction discrimination

Patient SL reported to have guessed the motion direction (upward | downward) of gratings in 90% of the trials and to have seen the motion direction in the remaining 10% of the trials. Under the unaware condition (i.e., “guessed” reports), SL’s accuracy in motion direction discrimination (55.28%) was significantly higher than chance level (*p* < 0.05), demonstrating implicit processing of motion direction of the bars. Conversely, under the aware condition (i.e., “seen” reports), SL’s accuracy in motion direction discrimination (58%) was not significantly higher than chance level (*p* = 0.189), demonstrating no ability to discriminate motion direction of bars under the aware condition. This lack of significance could, however, be related to a lack of power in the statistical analysis as the number of trials (*n* = 32) responded to be seen by SL is very small.

### Discussion

In the present experiment, patient SL was presented with a large variety of stimuli and asked to discriminate a stimulus feature in a 2AFC task while reporting whether or not she consciously perceived the stimulus using a binary measure. With the exception of contrast, patient SL reported to have guessed every feature in almost all the trials. Under this “guessing” condition, SL was nonetheless able to reach an accuracy level higher than chance for the discrimination of orientation, color, contrast, and (real) motion features of the stimuli. By using a binary measure to assess awareness, patient SL can, thus, be classified as a type 1 blindsight patient: visual processing in the absence of perceptual awareness.

In the next experiment, we tested SL’s visual abilities again within her blind visual field but changed how she reported awareness. Instead of a binary measure, we used a graded measure, the PAS (Overgaard et al., 2008).

## Experiment 2

### Materials and Methods

#### Participants

Patient SL was tested also in this experiment. In addition, we collected a sample of 10 healthy volunteers (aged 22–29 years) as a control group. They were all right-handed and all had normal or corrected-to-normal visual acuity with no history of neurological and psychiatry disorders.

All participants signed the informed consent prior to participating in the study and they were free to withdraw at any time. The study was approved by the local Ethics Committee and conducted in accordance with the 2013 Declaration of Helsinki.

#### Apparatus and Stimuli

Apparatus and stimuli were the same as in Experiment 1 with a few additions. In addition to stimulus feature used in Experiment 1 (high discriminability stimuli), we added another condition (low discriminability stimuli) for each of the five features tested: *orientation* (vertical | horizontal Gabor patches. Spatial frequency = 14 c/°. Luminance = 4.89 cd/m^2^), *color* (red | green Gaussian circles. Color coordinates: red: *x* = 0.4050, *y* = 0.3849; green: *x* = 0.3826, *y* = 0.4171. Luminance = 0.27 cd/m^2^), *contrast* (light | dark gray circles. Luminance: light gray = 5.02 cd/m^2^; dark gray = 4.75 cd/m^2^. Weber contrast = 0.03) and *direction of apparent* and *real motion* (upward | downward gratings and single bars. Speed of motion: grating = 66.66 °/s; bar = 33.33 °/s. Mean luminance: 4.89 cd/m^2^). The parameters of the low discriminability stimuli were selected on the basis of the results obtained in the group of healthy participants showing that their performance was at chance level. This additional condition served as a control condition in which chance-level performance was expected in patient SL. Moreover, to have the possibility to calculate criterion and sensitivity measures, catch trials were added. As in Experiment 1, patient SL was informed of the stimulus types. More specifically, in Experiment 2, she was told that in 20% of the trials the stimulus would not be presented. For both actual stimulus and catch trials, if feature discrimination was not possible, SL was asked to guess the feature of the stimulus. Both the blind and the intact visual fields were tested in SL while only the right visual field was tested in the group of healthy volunteers. For SL’s blind visual field, each experimental session, in which only one feature was tested, was composed of eight blocks of 100 trials each (20 trials per stimulus type and degree of discriminability plus 20 catch trials), for a total of 800 trials (e.g., for *orientation* 160 vertical and 160 horizontal Gabor patches at high discriminability, 160 vertical and 160 horizontal Gabor patches at low discriminability plus 160 catch trials). For SL’s intact visual field and the right visual field of healthy participants, we administered four blocks of 50 trials (10 trials per stimulus type and degree of discriminability plus 10 catch trials) for a total of 200 trials.

#### Experimental Procedure

The experimental procedure was the same as in Experiment 1 (**Figure 1C**) with the exception of how SL was to report awareness of the stimulus. In this experiment, instead of using a binary (seen | guess) forced-choice response, SL was asked to rate the clarity of her visual experience in her blind visual field according to the PAS developed by Ramsøy and Overgaard (2004). The PAS is composed of a four-level rating of the clearness of the visual experience: 0 = no visual experience; 1 = brief glimpse; 2 = almost clear visual experience; and 3 = clear visual experience. The actual meaning of each level was fully explained and discussed with the patient. To be certain that no misunderstanding could occur, we conducted a training session prior to the real experiment, frequently interrupting the patient to thoroughly discuss about her actual rating of the visual experience (Sandberg et al., 2013). Specifically, we stressed the distinction between level of confidence (as usually measured by confidence rating scales) and clearness of the visual experience as measured by the PAS (Sandberg et al., 2013). At the end of the training session, SL reported to have used PAS levels higher than zero (i.e., 1, 2, and 3) in occurrence with visual experiences of the stimuli and to scale these levels according with the clarity of her visual experiences.

As we expected that healthy controls would be able to detect the presence of the stimuli, they were not asked to rate the clarity of their visual experience but only to perform the 2AFC task with respect to the specific feature they were requested to discriminate.

#### Statistical Analysis

To assess whether the performance of healthy participants and patient SL in her intact visual field was significantly higher than chance level (50%) in detecting a specific stimulus feature we adopted the one-tailed binomial test. A total of 20 of one-tailed binomial tests (five feature discriminations by two levels of discriminability for the control group and patient SL) were performed. Moreover, to detect possible response biases toward a specific level of a feature, we analyzed the binomial distribution of the responses given with catch trials.

Given that with catch trials no *a priori* hypothesis on the direction of the difference can be made, we adopted the two-tailed binomial test to assess the presence of a response bias. The same analysis was performed on the data obtained with SL for stimuli presented in her blind field. The only difference is that for each stimulus feature, trials were classified off-line on the basis of the PAS responses, and the percent accuracy of feature discrimination was calculated for each of the four levels of the scale. The data were then entered in one-tailed or two-tailed binomial tests depending on the presence/absence of the visual stimulus as described above. Moreover, signal detection theory (SDT; Green and Swets, 1966) measures *d′* and *c* were used to assess sensitivity and response criterion, respectively, for each stimulus feature. Hit rate was defined as the trials in which the stimulus was presented and the PAS response was not zero (i.e., PAS = 1, 2, and 3). False alarms were defined as the trials in which the stimulus was not presented (catch trials) and the PAS response was not zero. Statistical significance was measured by means of receiver operating characteristic (ROC) curves. As a measure of goodness-of-fit, sensitivity (true positive), and specificity (true negative) were computed, and a ROC curve was generated. We calculated sensitivity and specificity pairs and plot sensitivity on the *y*-axis by (1-specificity) on the *x*-axis in order to create the ROC curve. The non-parametric (distribution-free) method to calculate the area under the curve (AUC) was used. AUC values range from 0.5 and 1.0 with larger values indicative of better fit. See Azzopardi and Cowey (1997) and Van den Stock et al. (2014) for more details on the procedure.

### Results

#### Healthy Participants: Accuracy

Healthy participants performed significantly (*p* < 0.001) above chance with high discriminability stimuli in discriminating orientation (98.63%), color (97.75%), contrast (99%), apparent (99.13%), and real (99.88%) motion. With low discriminability stimuli, they instead have an accuracy at chance level (lowest *p* > 0.1) for orientation (51.38%), color (51.75%), and apparent motion (56.88%) while they could discriminate contrast (76.25%) and real motion (63.13%) with an accuracy higher than chance (*p* < 0.05). In the same vein, they do not show any response bias with catch trials for orientation (49.50%), color (51.25%), and apparent motion (50.25%) while they showed a tendency for a response bias for contrast (57.25%, *p* = 0.113) and real motion (58.75%, *p* = 0.089).

Taken together, these results show that healthy participants performed above chance with high discriminability stimuli and at chance level with low discriminability stimuli (with the exception of contrast and real motion discrimination, for which, however, a possible confound of the presence of a response bias needs to be taken into account).

#### SL’s Intact Visual Field: Accuracy

Patient SL performed significantly (*p* < 0.001) above chance with high discriminability stimuli presented in her intact visual field in discriminating orientation (100%), color (100%), contrast (100%), apparent (97.50%), and real (100%) motion. With low discriminability stimuli SL showed an accuracy at chance level (lowest *p* > 0.6) for orientation (50%), color (52.50%), and apparent (52.50%) and real (53.75%) motion while she could discriminate contrast (87.50%) with an accuracy higher than chance (*p* < 0.001). As for catch trials, she did not show any response bias (lowest *p* > 0.3) for orientation (45%), color (50%), and apparent (57.50%) and real (52.50%) motion while she had a response bias (*p* < 0.001) for contrast discrimination (32.50%). As for healthy participants, these data show that in her intact visual field SL performed above chance with high discriminability stimuli and at chance level with low discriminability stimuli (with the exception of contrast discrimination, for which, however, the presence of a response bias need to be considered).

#### SL’s Blind Visual Field: Accuracy as a Function of the PAS

**Figures 1G,H** show SL’s rate of detection and accuracy in discriminating stimulus features as a function of her reports on the PAS. Statistical analysis is reported separately for each stimulus feature.

##### Orientation discrimination

In her blind field, SL reported no visual experience (PAS = 0) of the orientation (vertical | horizontal) for high discriminability stimuli in 39.50% of the trials and to have seen a brief glimpse (PAS = 1) of the stimulus in the remaining 60.50% of the trials. She never responded 2 or 3 on the PAS, indicating lack of any clear visual experience of the stimulus. Under the unaware condition (i.e., “no visual experience” reports; PAS = 0) SL’s accuracy (55.11%) was not significantly higher than chance level (*p* = 0.212), demonstrating no implicit processing of stimulus orientation. Similarly, SL’s accuracy in orientation discrimination (53.32%) was not significantly higher than chance level (*p* = 0.333) when she reported to have perceived a brief glimpse of the stimulus (PAS = 1), demonstrating that this level of awareness of the stimulus was not enough to discriminate stimulus orientation.

With stimuli at low discriminability presented in her blind visual field, SL reported no visual experience (PAS = 0) of the orientation (vertical | horizontal) of the stimuli in 24.14% of the trials and to have seen a brief glimpse (PAS = 1) of the stimulus in the remaining 75.86% of the trials. She never responded 2 or 3 on the PAS, indicating no clear/almost clear visual experience of the stimulus. Under the unaware condition (i.e., “no visual experience” reports; PAS = 0), SL’s accuracy in orientation discrimination (51.68%) was not significantly higher than chance level (*p* = 0.123) demonstrating no implicit processing of stimulus orientation. In the same vein, SL’s accuracy (49.75%) was not significantly higher than chance level (*p* = 0.475) when she reported to have perceived a brief glimpse of the stimulus (PAS = 1) demonstrating again that this level of awareness of the stimulus was not enough to discriminate stimulus orientation.

##### Color discrimination

Patient SL reported no visual experience (PAS = 0) of the color (red | green) of highly discriminable stimuli in 41.56% of the trials and to have seen a brief glimpse (PAS = 1) of the stimulus in the remaining 58.44% of the trials. She never responded with 2 or 3 on the PAS indicating that she did not have an almost clear or clear visual experience of the stimulus. Under the unaware condition (i.e., “no visual experience” reports; PAS = 0) SL’s accuracy in color discrimination (50.16%) was not significantly higher than chance level (*p* = 0.177) demonstrating no implicit processing of stimulus color. Similarly, SL’s accuracy (51.16%) was not significantly higher than chance level (*p* = 0.112) when she reported to have perceived a brief glimpse of the stimulus (PAS = 1) demonstrating that this level of awareness of the stimulus was not enough to discriminate stimulus color.

For stimuli of low discriminability presented in her blind visual field, SL reported no visual experience (PAS = 0) of the color (red | green) of the stimuli in the 8.13% of trials and to have seen a brief glimpse (PAS = 1) of the stimulus in the remaining 91.87% of trials (she never responded 2 or 3 at the PAS, indicating lack of any clear visual experience of the stimulus). Under the unaware condition (i.e., “no visual experience” reports; PAS = 0), SL’s accuracy in color discrimination (54.50%) was not significantly higher than chance level (*p* = 0.232) demonstrating no implicit processing of stimulus color. Similarly, SL’s accuracy (47.22%) was not significantly higher than chance level (*p* = 0.475) when she reported to have perceived a brief glimpse of the stimulus (PAS = 1) demonstrating again that this level of awareness of the stimulus was not enough to discriminate stimulus color.

##### Contrast discrimination

Patient SL reported no visual experience (PAS = 0) of the contrast (light | dark gray) of highly discriminable stimuli only in 0.94% of the trials (due to the low percentage, accuracy was not analyzed at this level of the PAS), to have seen a brief glimpse (PAS = 1) of the stimulus in 45.31% of the trials and to have perceived the stimulus almost clearly (PAS = 2) in the remaining 53.75% of the trials (she never responded 3 on the PAS, indicating no clear visual experience of the stimulus). SL’s accuracy in contrast discrimination was significantly higher than chance both when she reported a brief glimpse (PAS = 1: 65.51%, *p* < 0.001) and when she perceived the stimulus almost clearly (PAS = 2: 76.79%, *p* < 0.001), demonstrating that these levels of awareness of the stimulus enabled SL to discriminate stimulus contrast.

With stimuli at low discriminability presented in her blind visual field, SL reported no visual experience (PAS = 0) of the contrast (light | dark gray) of the stimuli in 78.75% of the trials and to have seen a brief glimpse (PAS = 1) of the stimulus in the remaining 21.25% of the trials (she never responded 2 or 3 on the PAS, indicating lack of any clear visual experience of the stimulus). Under the unaware condition (i.e., “no visual experience” reports; PAS = 0), SL’s accuracy in contrast discrimination (50.05%) was not significantly higher than chance level (*p* = 0.475) demonstrating no implicit processing of stimulus contrast. Similarly, SL’s accuracy (56.86%) was not significantly higher than chance level (*p* = 0.358) when she reported to have perceived a brief glimpse of the stimulus (PAS = 1), demonstrating again that this level of clearness of the stimulus was not enough to discriminate stimulus contrast.

##### Grating – (apparent) motion direction discrimination

Patient SL reported no visual experience (PAS = 0) of the motion direction (upward | downward) for high discriminability stimuli in the 59.56% of trials, to have seen a brief glimpse (PAS = 1) of the stimulus in 39.50% of the trials and to have seen the stimulus almost clearly (PAS = 2) only in 0.94% of the trials (due to the low percentage, accuracy was not analyzed at this level of the PAS). She never responded 3 on the PAS indicating that she never had a clear visual experience of the stimulus. Under the unaware condition (i.e., “no visual experience” reports; PAS = 0) SL’s accuracy in motion direction discrimination (52.80%) was not significantly higher than chance level (*p* = 0.306), demonstrating no implicit processing of stimulus motion direction. Similarly, SL’s accuracy (48.44%) was not significantly higher than chance level (*p* = 0.500) when she reported to have perceived a brief glimpse of the stimulus (PAS = 1) demonstrating that this level of awareness of the stimulus was not enough to discriminate stimulus motion direction.

With stimuli at low discriminability presented in her blind visual field, SL reported no visual experience (PAS = 0) of the discrimination of motion (upward | downward) of the stimuli in 67.50% of the trials, to have seen a brief glimpse (PAS = 1) of the stimulus in 23.19% of the trials and to have seen the stimulus almost clearly (PAS = 2) only in 0.31% of the trials (due to the low percentage, accuracy was not analyzed at this level of the PAS). She never responded 3 on the PAS, indicating that she never had a clear visual experience of the stimulus. Under the unaware condition (i.e., “no visual experience” reports; PAS = 0) SL’s accuracy in motion direction discrimination (54.11%) was not significantly higher than chance level (*p* = 0.188), demonstrating no implicit processing of stimulus motion direction. In the same vein, SL’s accuracy (57.38%) was not significantly higher than chance level (*p* = 0.500) when she reported to have perceived a brief glimpse of the stimulus (PAS = 1) demonstrating again that this level of awareness of the stimulus was not enough to discriminate stimulus motion direction.

##### Bar – (real) motion direction discrimination

Patient SL reported no visual experience (PAS = 0) of the direction of motion (upward | downward) for high discriminability stimuli in 33.33% of the trials and to have seen a brief glimpse (PAS = 1) of the stimulus in the remaining 66.67% of the trials (she never responded 2 or 3 on the PAS, indicating that she never had any clear visual experience of the stimulus). Under the unaware condition (i.e., “no visual experience” reports; PAS = 0) SL’s accuracy in motion direction discrimination (47.40%) was not significantly different from chance level (*p* = 0.500) demonstrating no implicit processing of stimulus motion direction. Similarly, SL’s accuracy in motion direction discrimination (50.64%) was not significantly higher than chance level (*p* = 0.419) when she reported to have perceived a brief glimpse of the stimulus (PAS = 1) demonstrating that this level of awareness of the stimulus was not enough to discriminate stimulus motion direction.

With stimuli at low discriminability presented in her blind visual field, SL reported no visual experience (PAS = 0) of the direction of motion (upward | downward) of the stimuli in 59.56% of the trials, to have seen a brief glimpse (PAS = 1) of the stimulus in the remaining 40.44% of the trials (she never responded 2 or 3 on the PAS), indicating that she never had any clear visual experience of the stimulus. Under the unaware condition (i.e., “no visual experience” reports; PAS = 0) SL’s accuracy in motion direction discrimination (52.23%) was not significantly higher than chance level (*p* = 0.213) demonstrating no implicit processing of stimulus motion direction. Similarly, SL’s accuracy in motion direction discrimination (49.16%) was not significantly higher than chance level (*p* = 0.363) when she reported to have perceived a brief glimpse of the stimulus (PAS = 1), demonstrating again that this level of awareness of the stimulus was not enough to discriminate stimulus motion direction.

##### Catch trials

With catch trials, SL reported to have no experience (PAS = 0) of the stimulus being presented in almost all the catch trials (*orientation*: 98.75% trials; *color*: 94.38%; *contrast*: 70%; *apparent direction of motion*: 80.63%; *real direction of motion*: 96.88%), thus demonstrating the ability to discriminate between the presence and the absence of the stimulus for all the features to discriminate. Moreover, she did not show any response bias in any of the different features tested (*orientation*: *p* = 0.385; *color*: *p* = 0.412; *contrast*: *p* = 0.333; *apparent direction of motion*: *p* = 0.813; *real direction of motion*: *p* = 0.813).

#### SL’s Blind Visual Field: Signal Detection Theory Analysis as a Function of the PAS

Given that SL’s performance was above chance only with high discriminability stimuli (and only these stimuli were used in Experiment 1), sensitivity and criterion were calculated only for these features. Results showed that sensitivity was high for all the stimulus features to be discriminated: orientation (PAS = 1: *d′* = 2.508), color (PAS = 1: *d′* = 1.800), contrast (PAS = 1: *d′* = 2.753; PAS = 2: *d′* = 4.251), apparent (PAS = 1: *d′* = 0.626), and real (PAS = 1: *d′* = 1.314) motion. Moreover, the area under the ROC curve was significant (*p* < 0.001) for all the conditions: orientation (PAS = 1: 0.773), color (PAS = 1: 0.743), contrast (PAS = 1: 0.884; PAS = 2: 0.982), apparent (PAS = 1: 0.606), and real (PAS = 1: 0.712) motion. Importantly, however, for almost all the features SL showed a response bias toward reporting to have seen the stimulus (orientation: *c* = 0.988; color: *c* = 0.687; apparent motion: *c* = 0.570; real motion: *c* = 0.226), even just as a brief glimpse, thus possibly lowering the reliability of the level of accuracy found for those stimuli (Azzopardi and Cowey, 1998). Conversely, SL showed an opposite response bias for contrast discrimination, (i.e., the tendency to report not having seen the stimulus) when she reported a brief glimpse (PAS = 1: *c* = -0.672) and no bias when she reported an almost clear visual experience (PAS = 2: *c* = 0.009).

### Discussion

In the present experiment, patient SL was presented with the same variety of stimuli as in Experiment 1 and asked to discriminate a stimulus feature while reporting whether or not she consciously perceived the stimulus using the PAS. Patient SL reported to have guessed the features of stimuli of high discriminability in about one-third of the trials. In contrast to the results of the previous experiment, when “guessing,” SL did not perform better than chance in any of the stimulus features. In contrast, she performed above chance only for contrast discrimination and when reported to have been aware, thus indicating a positive relationship between accuracy and the clarity of visual qualia. Interestingly, SL could discriminate the presence/absence of the stimulus for all the features tested, as she almost never reported seeing the stimulus when catch trials were presented. Moreover, the only condition in which SL demonstrated a high sensitivity with no bias in reporting having seen the stimulus was that of contrast discrimination, which is the condition in which SL performed above-chance level under the “aware” condition. Taken together, these data demonstrate that when using the PAS to assess awareness, patient SL cannot be classified as a type 1 blindsight patient but as a patient with conscious vision.

## General Discussion

In this study, we assessed the visual abilities within the blind field of one hemianopic patient. In two different experiments, we asked the patient to discriminate several features of visual stimuli briefly presented in her impaired visual field and to report visual awareness by means of binary (yes–no response, Experiment 1) or graded (four-level PAS, Experiment 2) measures. In Experiment 1, patient SL demonstrated type 1 blindsight (above-chance accuracy without acknowledged awareness) for orientation, color, contrast, and real motion discrimination. However, when asked to rate her perceptual experience with the PAS, her blindsight disappeared. In Experiment 2, indeed, she performed with above-chance accuracy for contrast discrimination only when she reported to have seen the stimuli, thus showing conscious, although degraded, vision, instead of pure blindsight. Taken together, the results of the two experiments show that patient SL’s performance cannot be interpreted as type 1 or type 2 blindsight as she experienced visual qualia in her “blind” field. Instead, SL’s visual abilities within her “blind” visual field can be better ascribed to a conscious experience though of a very different nature to that of normal vision.

Interestingly, in accordance with the findings by Overgaard et al. (2008), SL’s threshold to acknowledge conscious vision changed depending on the way awareness was assessed. When asked to assess her awareness of the stimuli by using a binary measure, she reported a mean percentage of acknowledged awareness in only 11.44% of the trials while when using the PAS, she reported (most of the times as a brief glimpse) to have seen the same stimuli in 65.02% of the trials. This evidence raises the possibility, already put forward elsewhere (Overgaard, 2011), that when asked to report whether they see a stimulus with a binary yes/no response, patients might be reluctant to acknowledge awareness for the kind of visual experience they have in their impaired visual field, thus setting their threshold too high and, as a consequence, increasing the number of false negatives of awareness by reporting conscious experience as unconscious. However, when patients are given a fine scale to categorize their visual experience of phenomenological properties, they tend to assess what they previously would have classified as “unseen” instead as having had the experience of brief glimpse if not as an almost clear visual experience. This possibility is in accord with several findings present in literature showing that visual processing within the impaired visual field is different from normal vision (Azzopardi and Cowey, 1998; Stoerig and Barth, 2001; Cowey, 2010). If conscious experience is characterized by different levels of clarity, a finer scale able to better characterize subtly different perceptual experiences is preferable as it might be more suitable to disentangle genuine forms of blindsight from degraded but conscious vision.

A final important point deserving some considerations relates to the possibility for patients with a complete lesion to V1 to experience conscious visual qualia. The clearest example of conscious vision in absence of V1 is illustrated by the Riddoch syndrome (Riddoch, 1917) in which patients are conscious of motion in their “blind” visual field despite denying having seen any other feature of the stimulus. The presence of visual qualia in patients with a lesion to V1 has two main important implications. First, the area of the visual field represented by the lesioned portion of V1 cannot be considered as totally blind (Cowey, 2010), as conscious experience is still possible, though of a different nature of normal vision. Second, the fact that a complete lesion to V1 does not completely abolish conscious vision, at least the crude type of consciousness sufficient to report simple visual qualia (Zeki and Ffytche, 1998), implies that V1 is not necessary for conscious vision (Ffytche and Zeki, 2011). To be tenable, however, V1 lesion needs to be complete (Fendrich et al., 1992). In this paper, we studied a patient whose lesion was well documented to be complete. Indeed, fMRI evidence (Celeghin et al., 2015b) showed no BOLD activity in her left V1 during visual presentation. Moreover, direct TMS to her left V1 (Mazzi et al., 2014; Bagattini et al., 2015), at an intensity well above threshold, did not result in any visual percepts. Although the activity in SL’s V1 was totally abolished, she experienced a certain level of awareness of the stimulus features she was presented with. The exact areas that might subserve conscious visual processing in absence of V1 is still unknown. It has been proposed that either subcortical pathways bypassing V1 (Schoenfeld et al., 2002), or prestriate cortex (Zeki and Ffytche, 1998) or areas along the dorsal stream (Goodale and Milner, 1992), more specifically the intraparietal sulcus (Mazzi et al., 2014; Silvanto, 2014; Bagattini et al., 2015), could be the neural correlates of these conscious percepts. These accounts are not mutually exclusive and a thoughtful discussion on this topic goes beyond the scope of this paper. What is important to stress here is that the present paper reports additional evidence of a patient with a complete lesion to V1 who, nonetheless, retains some kind of awareness of the stimuli presented to her impaired visual field, thus implying that V1 is not necessary for visual awareness, despite its importance for normal vision (Silvanto, 2015).

## Conclusion

The present data show that a finer scale to assess perceptual experience can more accurately identify conscious vision in hemianopic patients who would have been otherwise diagnosed erroneously as having type 1 blindsight with a dichotomous measure. Consequently, graded measures to assess awareness in hemianopic patients need to be used to better differentiate genuine forms of blindsight from degraded conscious vision, as they are clearly different phenomena (Azzopardi and Cowey, 1997; Marzi et al., 2004; Weiskrantz, 2009).

## Author Contributions

CM and CB designed and conducted the experiments and analyzed data. SS conceived the study, designed the experiments, and wrote the manuscript. All authors discussed the results and commented on the manuscript at all stages.

## Conflict of Interest Statement

The authors declare that the research was conducted in the absence of any commercial or financial relationships that could be construed as a potential conflict of interest.
